# Effect of Hataedock Treatment on Epidermal Structure Maintenance through Intervention in the Endocannabinoid System

**DOI:** 10.1155/2020/3605153

**Published:** 2020-01-22

**Authors:** Hee-Yeon Kim, Sang-hyun Ahn, In-Jun Yang, Sun-Young Park, Kibong Kim

**Affiliations:** ^1^Department of Korean Pediatrics, Pusan National University Korean Medicine Hospital, Geumo-ro 20, Mulgeum-eup, Yangsan-si, Gyeongsangnam-do 50612, Republic of Korea; ^2^Department of Korean Pediatrics, School of Korean Medicine, Pusan National University, Pusandaehak-ro 49, Mulgeum-eup, Yangsan-si, Gyeongsangnam-do 50612, Republic of Korea; ^3^Department of Anatomy, College of Korean Medicine, Semyung University, Semyung-ro 65, Jecheon-si, Chungcheongbuk-do 27136, Republic of Korea; ^4^Department of Physiology, College of Korean Medicine, Dongguk University, Dongdae-ro 123, Gyeongju-si, Gyeongsangbuk-do 38066, Republic of Korea; ^5^Department of Physiology, College of Korean Medicine, Semyung University, Semyung-ro 65, Jecheon-si, Chungcheongbuk-do 27136, Republic of Korea

## Abstract

The aim of this study was to investigate the efficacy of Hataedock (HTD) on skin barrier maintenance through the endocannabinoid system (ECS) intervention in *Dermatophagoides farinae*-induced atopic dermatitis (AD) NC/Nga mice. Douchi (fermented *Glycine max* Merr.) extracts prepared for HTD were orally administered to NC/Nga mice at a 20 mg/kg dose. Then, *Dermatophagoides farinae* extract (*Df*E) was applied to induce AD-like skin lesions during the 4th–6th and 8th–10th weeks. Changes in the epidermal structure of the mice were observed by histochemistry, immunohistochemistry, and TUNEL assay. The results showed that HTD significantly reduced the clinical scores (*p* < 0.01) and effectively alleviated the histological features. In the experimental groups, increased expression of cannabinoid receptor type (CB) 1, CB2, and G protein-coupled receptor 55 (GPR55) and distribution of filaggrin, involucrin, loricrin, and longevity assurance homolog 2 (Lass2) indicated that HTD maintained the epidermal barrier through intervening in the ECS. The expression of E-cadherin and glutathione peroxidase 4 (GPx4) was increased, and the levels of cluster of differentiation 1a (CD1A) were low. Moreover, the apoptosis of inflammatory cells was elevated. The production of phosphorylated extracellular signal-related kinase (p-ERK), phosphorylated c-Jun amino-terminal kinase (p-JNK), and phosphorylated mammalian target of rapamycin (p-mTOR) was low, and epidermal thickness was decreased. Besides, the expression levels of involucrin were measured by treating genistein, an active ingredient of Douchi extract, and palmitoylethanolamide (PEA), one of the ECS agonists. The results showed that genistein had a better lipid barrier formation effect than PEA. In conclusion, HTD alleviates the symptoms of AD by maintaining skin homeostasis, improving skin barrier formation, and downregulating inflammation, through ECS intervention.

## 1. Introduction

Atopic dermatitis (AD) is an inflammatory skin disease, highly relapsing, characterized by prorates, dryness, and erythematous eczema [1] and is also the initial stage of an atopic march that progresses to asthma and allergic rhinitis [2]. The pathogenesis of AD has not yet been elucidated and is thought to be caused by a combination of genetic, immunological, and environmental factors and skin barrier dysfunction [3]. Among them, skin barrier dysfunction has become the most important factor since the outside-inside hypothesis in the 1990s that damage to the skin barrier was the early mechanism of AD pathogenesis [4].

Levels of various keratinocyte differentiation markers, including filaggrin, involucrin, and loricrin, are low in AD lesions [5]. Moreover, levels of ceramide in stratum corneum (SC) are reduced [6]. Hallmarks of AD skin are epidermal hyperplasia resulting from increased epidermal proliferation and reduced differentiation [7] and spongiosis resulting from tissue remodeling [8]. These structural changes in the skin barrier disrupt skin homeostasis, preventing the skin from performing its normal barrier function.

The endocannabinoid system (ECS) is a biological system composed of cannabinoids (CBs) that regulate appetite, pain sensation, mood, and memory [9]. Recently, it was revealed that an increase or decrease of the ECS tones is associated with the various pathological conditions [10]. Temporarily altered activity of ECS reduces the symptoms of the body's compensatory response or slows disease progression. In other cases, activation of ECS can act as a pathogenic or reflect a defect in the body [11]. In particular, the ECS is associated with the regulation of cell growth, proliferation, immunity, and the inflammatory response involved in skin homeostasis [12]. Representative CBs, anandamide (N-arachidonoylethanolamine; AEA) and 2-arachidonoylglycerol (2-AG), which are produced locally in various cellular compartments of the skin, regulate the various cutaneous functions via binding to cannabinoid receptor type (CB)1 or CB2 [13]. In epidermal keratinocytes, activation of CB1 and CB2 suppresses cellular proliferation and differentiation [14], releases the inflammatory mediators [15], and induces apoptosis [16]. Additionally, CB1 suppresses the secretion of proinflammatory chemokines to help control skin inflammation [17]. In the hair follicle, activation of CB1 attenuates hair growth and proliferation, whereas promotes apoptosis and the regression phase [18]. In the sebaceous gland, activation of CB2 stimulates lipid formation and apoptosis [19]. Furthermore, various CBs inhibit sensory phenomena such as pain and itching via CB1 [20, 21]. ECS constitutively regulates the well-balanced proliferation and differentiation of skin cells, as well as immune and inflammatory responses. The fine-tuned changes in ECS might promote or alleviate skin diseases [13]. Therefore, the ECS is a microenvironmental control factor for maintaining skin homeostasis. Thus, the role of the ECS as a new therapeutic target for skin diseases has been highlighted [22].

In traditional Chinese medicine, inflammatory diseases such as asthma, rhinitis, and AD are thought to be due to heat syndrome [23]. Therefore, inflammatory diseases are treated with heat-clearing herbal medicine to remove the accumulated heat in the body [24]. Fetal heat is caused by changes in the microimmune environment that affect fetal survival, such as in Th2-skewed conditions. Thus, fetal heat manifests as a variety of diseases in newborns by disrupting the homeostasis of the fetus, of which AD is the most common [25]. In Korean medicine, Hataedock (HTD), herbal extracts that are orally administered to neonates and infants, was used to clear fetal heat to prevent inflammatory diseases. Our previous studies have shown that HTD mitigates AD development due to fetal heat and controls Th2-skewed conditions [26, 27]. Moreover, our studies showed that lipid barrier formation in the epidermis is increased after the application of HTD [28].

Douchi (fermented *Glycine max* Merr.), one of the most commonly used herbs in HTD, is a kind of fermented soybean known as a herb that reduces heat by radiating the body heat [29]. Recently, many studies have been conducted on ECS modulators, especially soybean [30–32]. In particular, genistein, a modest fatty acid amide hydrolase (FAAH) inhibitor, can prevent proinflammatory response in a CB1/CB2-dependent manner [33]. In our previous study, we found that Douchi extract contains more genistein than raw soybeans [26].

HTD is a prophylactic treatment applied to neonates and infants before an inflammatory disease occurs and is not a treatment of inflammatory disease. However, there have been no studies on the mechanism by which HTD can prevent inflammatory diseases. Therefore, based on the results of inflammation relief and lipid barrier formation induced by HTD, we hypothesized that HTD intervenes in the ECS to maintain skin homeostasis and that the ECS maintains the epidermal structure by influencing microenvironmental control of the epidermis. Here, we investigated changes in the epidermal structure to confirm how HTD influences AD alleviation through ECS intervention.

## 2. Materials and Methods

### 2.1. Preparation of HTD Herbal Extracts

In this study, the Douchi (豆豉, fermented *Glycine max* Merr.) was purchased from Namyoung Pharm (Muju, Republic of Korea). Douchi is a black bean fermented for 5 days at 37–38°C in *Artemisia Apiacea Herba* and *Mori Folium* extract (1 : 1). The Douchi extract was prepared as follows: (1) 100 g of Douchi was boiled for 3 hours in 1 L of distilled water and then filtered, and (2) the filtrate was concentrated to 50 mL by using a rotatory vacuum evaporator and then freeze-dried to obtain 15 g of the extract (yield: 15.0%).

### 2.2. Experimental Animals and AD Induction

**Figure 1:**
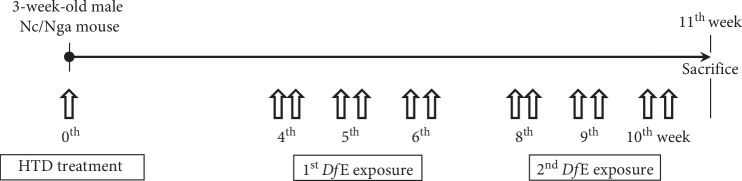
Experiment design. Prior to inducing AD-like skin lesion, FGT group was applied with Douchi (fermented *Glycine max* Merr.) extracts orally on days 1, 2, and 3. The mice were repeatedly induced by *Df*E at the 4^th^, 5^th^, 6^th^, 8^th^, 9^th^, and 10^th^ weeks. HTD: Hataedock and *Df*E: *Dermatophagoides farinae* extract.

In this study, we used 3-week-old male NC/Nga mice (14.3 ± 0.3 g each) purchased from Central Lab Animal Inc. (Seoul, Republic of Korea). The animals were kept under standard laboratory conditions (25°C and 12/12 h light/dark cycles) and standard food and water were freely provided. The mice were allocated randomly to 3 groups (*n* = 10 per group) as follows: the normal group (Ctrl group), AD-induced with no treatment group (AE group), and AD-induced with HTD treatment group (FGT group). In the FGT group, 3-week-old male NC/Nga mice were orally administered Douchi extracts at 20 mg/kg on the 1^st^, 2^nd^, and 3^rd^ days were used for HTD treatment. To induce AD-like skin lesions, the back skin regions of the mice were exposed and 1 mL of 5% sodium dodecyl sulfate (Sigma-Aldrich, St. Louis, MO, USA) was rubbed 20 times to remove the lipid lamella of the SC of each mouse. Then, *Dermatophagoides farinae* extract (*Df*E) (100 mg, Biostir Inc., Osaka, Japan) was administered at the 4^th^, 5^th^, and 6^th^ weeks and administered again at the 8^th^, 9^th^, and 10^th^ weeks. In the 11^th^ week, the mice were sacrificed with sodium pentobarbital. All animal experiments were approved by the Institutional Animal Care and Use Committee of Pusan National University (IACUC number: PNU-2015-0924). We followed the National Institutes of Health (NIH) Guide for the Care and Use of Laboratory Animals throughout this study. The overall experimental protocol is summarized in Figure 1.

### 2.3. Dermatitis-Induced Animal Model

To induce skin lesions, the back skin regions of the mice were exposed and 1 mL of 5% sodium dodecyl sulfate (Sigma-Aldrich, St. Louis, MO, USA) was rubbed 20 times to remove the lipid lamella of the SC of each mouse. The mice were allocated randomly to 4 groups (*n* = 10 per group) as follows: the normal group (Ctrl group), dermatitis-induced with no treatment group (DE group), dermatitis-induced with genistein 10 mg/kg treatment group (GT group), and dermatitis-induced with palmitoylethanolamide (PEA) 10 mg/kg treatment group (PT group). In the GT and PT groups, 6-week-old male BALB/c mice were orally administered genistein and PEA for 3 days.

### 2.4. Western Blotting

3D human skin keratinocytes were dispensed into the 6-well plate as 4 × 105 cells/well, cultured in 80% confluence, and then cultured for 24 hours after treated with reagents. After culture, the lysate was prepared by treating with RIPA buffer (Atto, Tokyo, Japan) and centrifuged at 12,000 rpm at 4°C for 10 minutes to recover the supernatant. After protein quantification was performed using the Bradford protein assay reagent, 50 *μ*g of proteins was separated by SDS-polyacrylamide gel (10%) and transferred to a polyvinylidene difluoride (PVDF) membrane at 190 mA for 80 minutes. The membrane was blocked in PBS containing 5% skim milk for 1 hour at 37°C and treated overnight with an anti-involucrin antibody (# ab24722, # ab53112, Cambridge, MA, USA). In addition, it was treated with HRP-conjugated anti-rabbit antibody at room temperature for 1 hour. After completion of the reaction, the membrane was developed using an enhanced chemiluminescence system (Bio-Rad Laboratories, Hercules, USA) and observed for protein expression on X-ray film.

Skin tissues were homogenized with ice-cold tissue extraction reagent (Thermo Fisher Scientific, Vienna, Austria) containing protease and phosphatase inhibitors (Atto, Tokyo, Japan) and centrifuged at 10,000 rpm for 20 minutes, and supernatants were collected. Amounts of proteins in samples were determined using Bradford protein assay reagent (Bio-Rad, Hercules, CA, USA). Proteins (50 *μ*g) were separated by 10% SDS-PAGE electrophoresis and transferred to polyvinylidene difluoride membranes (Merck Millipore, Carrigtwohill, Ireland), which were blocked with 5% skim milk in PBS for 2 hours at room temperature, and incubated with primary antibodies, followed by secondary antibody horseradish peroxidase-conjugated anti-IgG. Anti-involucrin was purchased from Santa Cruz Biotech (Paso Robles, CA, USA). All bands were detected by enhanced chemiluminescence (Bio-Rad, Hercules, CA, USA).

### 2.5. Evaluation of Clinical Symptoms

The morphologic severity of the dorsal skin was evaluated after 3 weeks and compared to the baseline. The evaluation items of the skin score are as follows: (1) erythema/hemorrhage, (2) scarring/dryness, (3) edema, and (4) excoriation/erosion were scored as 0 (none), 1 (mild), 2 (moderate), or 3 (severe). The sum of the individual scores was defined as the AD skin score [34]. Transepidermal water loss (TEWL) was measured for 30 seconds using a Vapometer device (Delfin Technologies, Finland) in accordance with the manufacturer's manual.

### 2.6. Angiogram

The capillary distribution was photographed at 4x magnification using a sharpen low-filter in Image-Pro Plus (Media Cybernetics, Rockville, USA) and then inverted (180–200 intensity range).

### 2.7. Tissue Processing and Histochemistry

After sacrificing the mice, the obtained dorsal skin was fixed with 10% NBF at room temperature for 24 hours, and the fixed skin tissues were embedded in paraffin. Then, skin samples at a thickness of 5 *μ*m were histochemically stained. To examine histological changes such as collagen fiber distribution and epithelial hyperplasia, we performed Masson's trichrome (M/T) staining. Toluidine blue staining was performed to investigate the distribution and morphological changes in the intercellular space of keratinocytes. To investigate the inflammatory changes in the epidermis, we performed phloxine-tartrazine (P/T) staining.

### 2.8. Immunohistochemistry

The skin samples were treated with proteinase K solution (20 *μ*g/mL) for 5 minutes at room temperature, and the proteolyzed skin samples were incubated in blocking serum (10% normal goat serum) for 4 hours. Then, the skin samples were incubated with primary antibodies (all antibodies used in the experiment were purchased from Santa Cruz Biotechnology, Dallas, TX, USA), including goat anti-interleukin- (IL-) 4 (IL-4; 1 : 100), goat anti-CB1 (1 : 100), goat anti-CB2 (1 : 100), goat anti-G protein-coupled receptor 55 (GPR55; 1 : 100), rabbit anti-filaggrin (1 : 100), rabbit anti-involucrin (1 : 50), rabbit anti-loricrin (1 : 50), goat anti-longevity assurance homolog 2 (Lass2; 1 : 100), goat anti-protein kinase C (PKC; 1 : 100), goat anti-E-cadherin (1 : 100), goat anti-glutathione peroxidase 4 (GPx4; 1 : 100), goat anti-cluster of differentiation 1a (CD1A; 1 : 100), goat anti-phosphorylated extracellular signal-related kinase (p-ERK; 1 : 100), goat anti-phosphorylated c-Jun amino-terminal kinase (p-JNK; 1 : 100), and goat anti-phosphorylated mammalian target of rapamycin (p-mTOR; 1 : 100) for 72 hours in a 4°C humidified chamber. Next, the samples were incubated with biotinylated rabbit anti-goat IgG (1 : 100), secondary antibody, at room temperature for 24 hours. Then, an avidin-biotin complex kit (Vector Lab, Burlingame, CA, USA) was used at room temperature for 1 hour. As a final step, the samples were treated with 0.05 M Tris-HCl buffer solution (pH 7.4) composed of 0.05% 3,3′-diaminobenzidine and 0.01% HCl and counterstained with hematoxylin.

### 2.9. TUNEL Assay

The terminal deoxynucleotidyl transferase-mediated dUTP-digoxigenin nick end labeling (TUNEL) assay was conducted using an in situ apoptosis detection kit (Apoptag, Intergen, New York, USA) to observe apoptosis. The skin samples were incubated with proteinase K solution (20 *μ*g/mL) for 5 minutes and treated with equilibration buffer solution for 5 seconds. Then, the samples were reacted with TdT enzyme (36 *μ*L TdT enzyme: 72 *μ*L reaction buffer). Subsequently, the samples were incubated in a humidified  chamber  at  37°C  for  1 hour and washed in the intensity stop/wash buffer for 10 minutes, and the reaction was terminated with anti-digoxigenin peroxidase and DAB for 1 hour. We then observed the counterstained sections with eosin using an optical microscope.

### 2.10. Image Analysis and Statistical Analysis

The results of the immunohistochemical and TUNEL assays were digitized by image analysis using Image-Pro Plus (Media Cybernetics). The data are presented as the mean ± standard error. The image analysis was performed at 400x magnification of randomly selected fields of each group. The statistical data were analyzed with SPSS software (SPSS 23, SPSS Inc., Chicago, IL, USA). The significance was verified by using a one-way analysis of variance (ANOVA) and Levene's (LSD) test with a significance level of *p* < 0.01.

## 3. Results

### 3.1. Alleviation of AD

**Figure 2:**
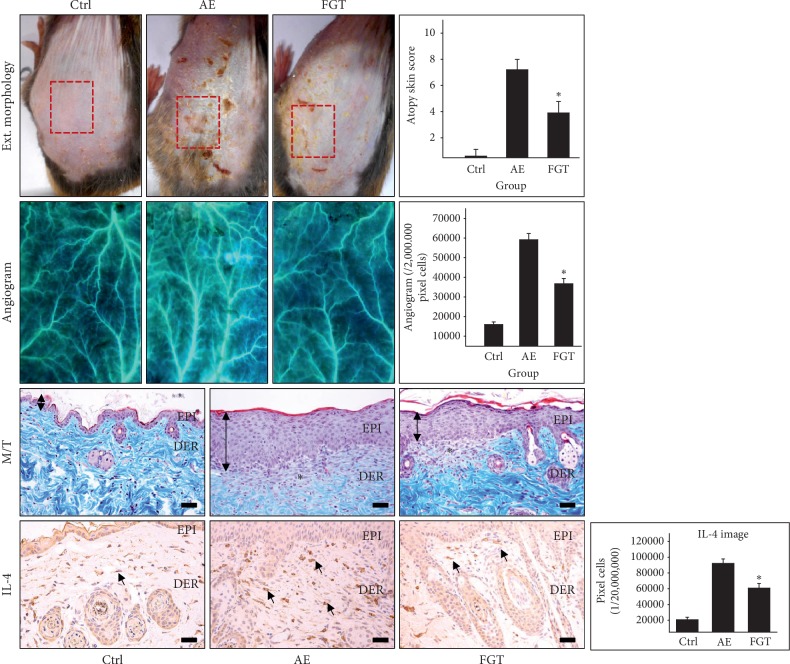
The alleviation effects on symptoms of AD. HTD relieved the AD-like skin lesions in the FGT group. The capillary distribution is increased in the AE group but decreased in the FGT group (4x) (*p* < 0.01). In M/T staining result, edema of papillary dermis is decreased in the FGT group, but increased in the AE group (*p* < 0.01) (M/T staining; asterisk, edema invoked region; bar size, 50 *μ*m). The IL-4-positive reactions (arrow indicates dark brown) were decreased in the AE group compared with the FGT group (*p* < 0.01) (immunohistochemistry; bar size, 50 *μ*m). ^*∗*^*p* < 0.01, compared with the AE group. Ctrl: normal, AE: AD-induced with no treatment, FGT: AD-induced with HTD treatment, EPI: epidermis, DER: dermis, and M/T: Masson's trichrome staining.

HTD-mediated alleviation of AD was estimated based on external morphology, clinical symptoms, AD skin scores, and angiogenesis. The external image of the AE group showed the most severe pathological features such as erythema, hemorrhage, scarring, dryness, edema, erosion, and excoriation. In contrast, HTD alleviated AD symptoms in the FGT group (Figure 2). The AD skin scores were evaluated at 7.2 ± 0.25 in the AE group and 3.9 ± 0.28 in the FGT group. Significant differences were found as the skin scores of the FGT group were decreased by 46% (*p* < 0.01) compared with those of the AE group (Figure 2).

To compare AD-associated angiogenesis, we conducted angiograms in the AE and FGT groups. The angiogenesis was 59,153 ± 996/20,000,000 pixels in the AE group, which was 271% (*p* < 0.01) higher than that of the Ctrl group. In contrast, the angiogenesis in the FGT group was 36,705 ± 861/20,000,000 pixels which were decreased by 38% (*p* < 0.01) compared with that of the AE group. The angiogram indicated that angiogenesis was more suppressed in the FGT group than in the AE group (Figure 2).

In addition, we used M/T staining to observe papillary dermal edema. The results showed that papillary dermal edema and capillary distribution were increased and that the distribution of collagen fibers in the dermis was decreased in the AE group. Moreover, the infiltration of inflammatory cells into the basement of the epidermis in the AE group was significantly increased. In contrast, papillary dermal edema was significantly decreased in the HTD treatment group (Figure 2).

One of the important features of AD, the expression of increased Th2 cytokines, was estimated by measuring IL-4-positive reactions in the cytoplasm of papillary dermal cells. IL-4-positive reactions in the AE group were increased by 345% (*p* < 0.01) compared with those of the Ctrl group. In contrast, the IL-4-positive reactions in the FGT group were decreased by 34% (*p* < 0.01) compared with those of the AE group. Significant differences were shown between all of the groups (*p* < 0.01) (Figure 2).

Overall, these results suggest that HTD alleviates the development of AD in NC/Nga mice.

### 3.2. Activation of the ECS

Recent studies revealed that the ECS is involved in the regulation of skin homeostasis by controlling cell growth, differentiation, and immune and inflammatory responses [13]. Thus, the inadequate function of the ECS is directly related to skin disorders.

**Figure 3:**
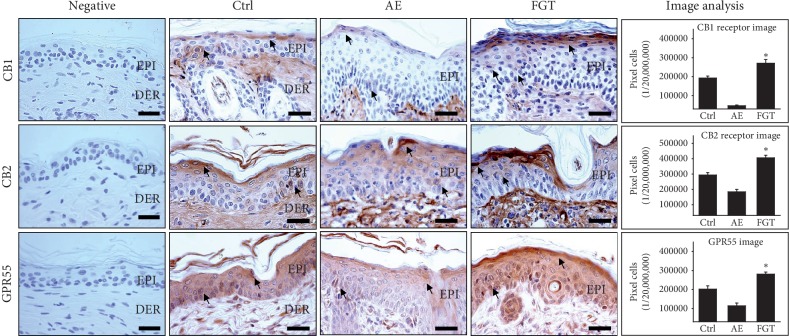
The ECS activation effects of HTD. HTD generates the ECS in the FGT group. The CB1, CB2, and GPR55 expression (arrow indicates dark brown) was significantly increased in the FGT group as compared with the AE group (*p* < 0.01) (bar size, 50 *μ*m). The data of CB1, CB2, and GPR55 image analysis showed the same results (*p* < 0.01). ^*∗*^*p* < 0.01, compared with the AE group. Ctrl: normal, AE: AD-induced with no treatment, FGT: AD-induced with HTD treatment, EPI: epidermis, and DER: dermis.

To confirm the activation of the ECS, we measured the expression levels of CB1, CB2, and GPR55 through immunohistochemical staining. Compared with the levels in the AE group, HTD significantly increased the expression levels of CB1, CB2, and GPR55. The levels of CB1 expression in the FGT group were increased by 476% (*p* < 0.01) compared with those of the AE group. The FGT group showed a 121% (*p* < 0.01) increase in CB2 expression compared with that of the AE group. In addition, the levels of GPR55 expression in the FGT group were increased by 147% (*p* < 0.01) compared to those in the AE group (Figure 3). These results suggest that HTD has the potential to suppress skin disorders through the intervention of ECS.

### 3.3. Regulation of the Lipid Barrier

The cornified cell envelope (CE), which is composed of filaggrin, involucrin, loricrin, and other proteins, is important in the skin barrier [35]. Moreover, recent studies revealed that reduced expression of involucrin and loricrin in AD could exacerbate AD [36]. These studies indicated that an abnormal epidermal barrier is not simply an epiphenomenon of AD but rather promotes inflammation in AD [37].

To determine the effect of HTD on the epidermal lipid barrier, we measured the levels of filaggrin, involucrin, and loricrin and the levels of PKC and Lass2, which are involved in epidermal lipid metabolism [38, 39].

The levels of filaggrin in keratohyalin granules in the SC were markedly reduced in the AE group, whereas the levels of filaggrin in the FGT group were increased by 91% (*p* < 0.01) compared with those in the AE group. Increased levels of involucrin were observed in the cornified layer of the SC in the FGT group. The levels of involucrin in the FGT group were increased by 210% (*p* < 0.01) compared with those of the AE group.

**Figure 4:**
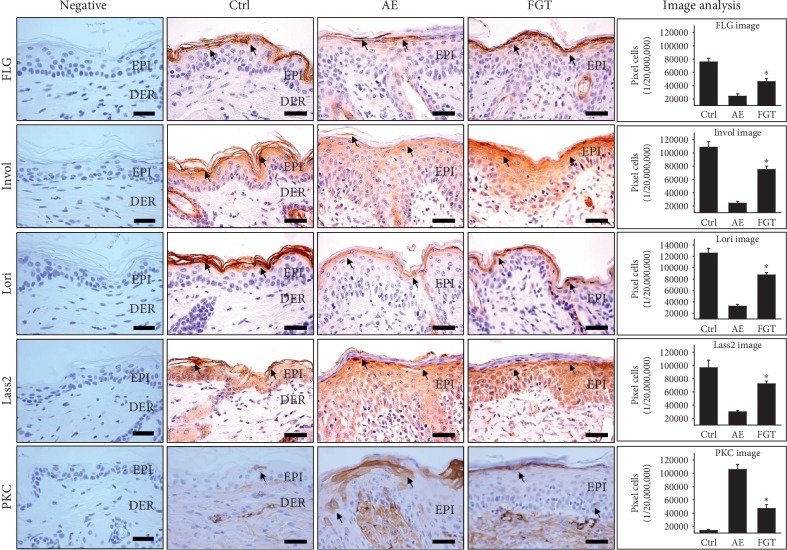
The lipid barrier regulatory effects of HTD. HTD repaired the lipid barrier in the FGT group. The filaggrin, involucrin, loricrin, and Lass2-positive reaction (arrow indicates dark brown) were significantly increased in the FGT group as compared with the AE group (*p* < 0.01) (bar size, 50 *μ*m). The PKC-positive reaction (arrow indicates dark brown) was remarkably reduced in the FGT group compared with the AE group (*p* < 0.01) (bar size, 50 *μ*m). ^*∗*^*p* < 0.01, compared with the AE group. Ctrl: normal, AE: AD-induced with no treatment, FGT: AD-induced with HTD treatment, EPI: epidermis, DER: dermis, FLG: filaggrin, Invol: involucrin, and Lori: loricrin.

Moreover, the levels of loricrin in the AE group were decreased by 74% (*p* < 0.01) compared with those of the Ctrl group. This decrease was not observed in the FGT group. The levels of loricrin in the FGT group were increased by 170% (*p* < 0.01) compared with those of the AE group (Figure 4).

Levels of Lass2, a ceramide synthase, were 29,821 ± 627/20,000,000 pixels which were markedly decreased by 69% (*p* < 0.01) compared with those of the Ctrl group. However, HTD increased the levels of Lass2 in the FGT group by 142% (*p* < 0.01) compared with those of the AE group. The levels of Lass2 were 72,258 ± 1315/20,000,000 pixels in the FGT group (Figure 4).

In the AE group, an increase in PKC-positive reactions was observed in damaged keratinocytes. However, this increase was not observed in the FGT group that was orally administered Douchi. The levels of PKC in the AE group were 105,789 ± 2451/20,000,000 pixels which were significantly increased by 664% (*p* < 0.01) compared with those of the Ctrl group. In the FGT group, the levels of PKC were 46,958 ± 1874/20,000,000 pixels which were significantly reduced by 56% (*p* < 0.01) compared with those of the AE group (Figure 4).

Thus, by modulating filaggrin, involucrin, loricrin, Lass2, and PKC expression, HTD might serve as a highly effective agent for improving lipid barrier formation.

### 3.4. Regulation of Epidermal Structure

**Figure 5:**
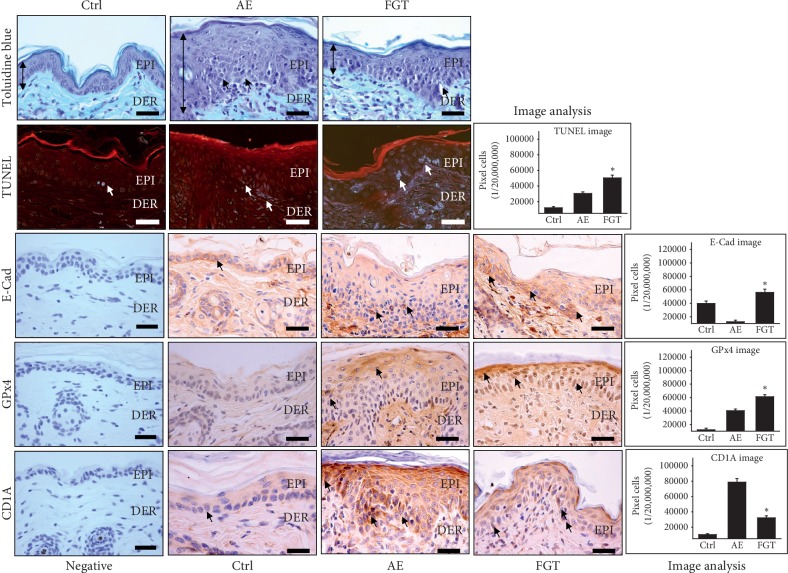
The epidermal structure regulatory effects of HTD. HTD improved the epidermal structures in the FGT group. In toluidine blue staining result, epidermal thickness (arrow (↕) indicates), infiltration lymphocytes (arrow indicates dark navy), and intercellular spaces (arrow indicates white) were increased in the AE group but decreased in the FGT group (*p* < 0.01) (toluidine blue staining; bar size, 50 *μ*m). The E-cadherin expression (arrow indicates dark brown) was decreased in the AE group, but significantly increased in the FGT group (*p* < 0.01) (bar size, 50 *μ*m). The GPx4 expression (arrow indicates dark brown) in the FGT group was remarkably increased compared with the AE group (*p* < 0.01) (bar size, 50 *μ*m). The CD1A expression (arrow indicates dark brown) was significantly increased but decreased in the FGT group (*p* < 0.01). In TUNEL assay, the apoptotic body (arrow indicates white-blue fluorescence) in the FGT group was remarkably increased compared to the AE group (TUNEL assay; bar size, 50 *μ*m). ^*∗*^*p* < 0.01, compared with the AE group. Ctrl: normal, AE: AD-induced with no treatment, FGT: AD-induced with HTD treatment, EPI: epidermis, DER: dermis, and E-Cad: E-cadherin.

Typically, epidermal thickness due to epidermal hyperplasia and spongiosis is observed in AD skin lesions [40]. The spongiosis, which expands the intercellular spaces in the epidermal basal lamina, is formed by the impairment of intercellular adhesions between the epidermal keratinocytes [41]. We performed a comparison of epidermal thickness between the AE and FGT groups through toluidine blue staining. The results showed that the intercellular spaces of the AE group were significantly increased compared to those of the Ctrl group. On the other hand, HTD reduced the intercellular spaces to a level comparable to that in the Ctrl group. In addition, the migration of lymphocytes and the distribution of capillaries in the dermal papilla of the AE group were significantly increased compared with those in the Ctrl group. In contrast, the extent of lymphocyte migration and distribution of capillaries were significantly decreased in the HTD treatment group (Figure 5). These findings indicate that the expansion of intercellular space causes the basement membrane to collapse and lymphocytes to migrate.

To examine the effect of HTD on the cell-cell junctions of the epidermal structure, we measured the levels of E-cadherin, which is an adherens junction protein. E-cadherin expression was markedly reduced in the AE group, whereas the levels of E-cadherin in the FGT group were increased by 347% (*p* < 0.01) as compared with those of the AE group. The levels of E-cadherin in the AE group were 12,552 ± 722/20,000,000 pixels which were markedly decreased by 68% (*p* < 0.01) compared with those of the Ctrl group. However, the levels of E-cadherin in the FGT group were 56,147 ± 1559/20,000,000 pixels, which were higher than those of the Ctrl group (Figure 5).

Recent, studies revealed that the expression of GPx4, an antioxidant enzyme, is increased by the induction of transcriptional response to oxidative stress in the skin of AD patients [42]. Moreover, GPx4 regulates apoptosis by releasing the apoptogenic proteins [43]. Thus, we examined the levels of GPx4 to investigate the protective effect of HTD against oxidative damage by oxidative stress. The levels of GPx4 in the AE group were 40,451 ± 798/20,000,000 pixels which were markedly increased by 235% (*p* < 0.01) compared with those of the Ctrl group. The expression of GPx4 in the FGT group was 61,253 ± 987/20,000,000 pixels, which was 51% (*p* < 0.01) higher than that of the AE group (Figure 5). These findings mean that HTD acts as a defense against oxidative stress by increasing the expression of GPx4 and contributes to maintaining cell homeostasis through the regulation of apoptosis.

CD1A is often used as a marker for evaluating the number of epidermal Langerhans cells [44]. Recent studies have reported that CD1A expression is increased in AD skin lesions compared to that of nonlesions [45]. Moreover, the disruption of the epidermal structure allows skin resident antigen-presenting cells such as Langerhans cells to capture environmental antigens [46]. Therefore, we tried to confirm the destruction of the epidermal structure through CD1A staining. In the AE group, an increase in CD1A expression was observed. The levels of CD1A in the AE group were 78,693 ± 1506/20,000,000 pixels which were significantly increased by 661% (*p* < 0.01) compared with those of the Ctrl group. On the other hand, the levels of CD1A in the FGT group were 32,167 ± 811/20,000,000 pixels which were significantly reduced by 59% (*p* < 0.01) compared with those of the AE group (Figure 5).

Furthermore, the TUNEL assay demonstrated that the HTD maintains the epidermal structure by promoting apoptosis between the stratum spinosum (SP) and stratum basale (SB). In the AE group, the number of apoptotic cells was 30,360 ± 682/20,000,000 pixels which were increased by 150% compared with that of the Ctrl group. However, the number of apoptotic cells in the FGT group was 50,373 ± 1119/20,000,000 pixels which were markedly increased by 66% (*p* < 0.01) in contrast to the increase in the AE group (Figure 5). These results indicate that HTD maintains the epidermal structure by inducing the apoptosis of inflammatory cells.

Thus, by modulating the intercellular space, expression of E-cadherin, GPx4, and CD1A, and apoptosis, HTD might serve as a highly effective therapy for improving epidermal structures.

### 3.5. Regulation of Epidermal Inflammation

To estimate the anti-inflammatory effects of HTD, we performed P/T staining and measured the expression levels of p-ERK, p-JNK, and p-mTOR.

**Figure 6:**
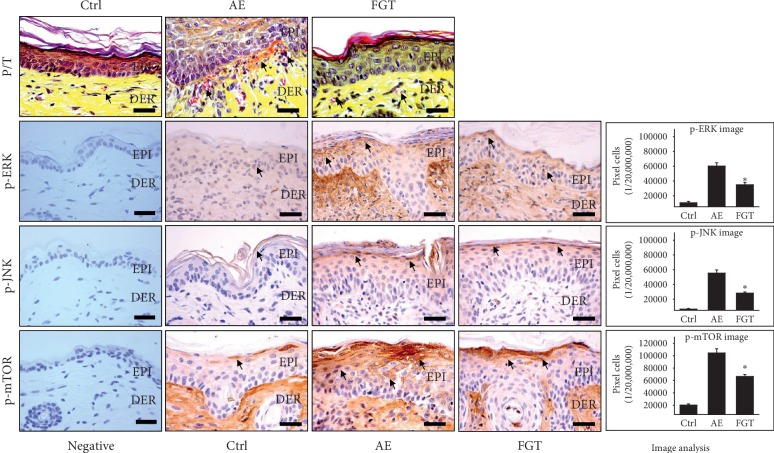
The epidermal inflammation regulatory effects of HTD. HTD reduced the epidermal inflammation in the FGT group. In P/T staining result, infiltration of inflammatory cells and capillary distributions (arrow indicates red) were increased in the AE group but decreased in the FGT group (*p* < 0.01) (P/T staining; bar size, 50 *μ*m). The p-ERK, p-JNK, and p-mTOR expressions (arrow indicates dark brown) were increased in the AE group, but significantly decreased in the FGT group (*p* < 0.01) (bar size, 50 *μ*m). ^*∗*^*p* < 0.01 compared with the AE group. Ctrl: normal, AE: AD-induced with no treatment, FGT: AD-induced with HTD treatment, EPI: epidermis, DER: dermis, and P/T: phloxine-tartrazine.

The results of P/T staining showed that the infiltration of inflammatory cells such as granulocytes and lymphocytes in the AE group was significantly increased compared to that of the Ctrl group. On the other hand, the infiltration of inflammatory cells after HTD was reduced to a level similar to that of the Ctrl group (Figure 6).

In the cell, inflammatory signals activate various signaling molecules, among which mitogen-activated protein kinases (MAPKs) are key signaling molecules that activate various transcription factors through phosphorylation [47]. MAPKs, including ERK and JNK, are involved in the activation of transcription factors including nuclear factor kappa-light-chain-enhancer of activated B cells (NF-κB) and activator protein 1 (AP-1), thereby increasing the secretion of inflammatory mediators [48]. Thus, the levels of p-ERK and p-JNK were evaluated to investigate the effect of HTD on the activation of phosphorylated MAPKs. The levels of p-ERK in the AE group were 80,166 ± 1399/20,000,000 pixels which were significantly increased by 665% (*p* < 0.01) compared with those of the Ctrl group. In contrast, the levels of p-ERK in the FGT group were 34,803 ± 1001/20,000,000 pixels which were significantly reduced by 57% (*p* < 0.01) compared with those of the AE group (Figure 6). The results of p-JNK expression were similar to the results for p-ERK. Compared with the levels in the Ctrl group, the p-JNK levels of the AE group were 55,413 ± 1355/20,000,000 pixels which were increased by 736%. After HTD, the p-JNK levels decreased. The p-JNK levels in the FGT group were 28,299 ± 482/20,000,000 pixels which were decreased by 49% (*p* < 0.01) compared with those of the AE group (Figure 6).

Recently, studies reported that inflammatory cytokines such as tumor necrosis factor-*α* (TNF-*α*), IL-1*β*, and IL-17A induce mTOR activation to promote the epidermal proliferation and reduce the expression of epidermal differentiation markers [49]. Moreover, topical application of the mTOR inhibitor rapamycin to AD skin lesions resulted in decreased inflammatory cell infiltration and serum IgE levels, suggesting that inhibition of mTOR signaling suppresses AD [50]. Therefore, we performed an immunohistochemical analysis of expression of p-mTOR, the activation form of mTOR, to determine the effects of HTD. The expression of p-mTOR in the AE group was 104,341 ± 2111/20,000,000 pixels which were significantly increased by 417% (*p* < 0.01) compared with that of the Ctrl group. In contrast, p-mTOR expression in the FGT group was 66,372 ± 977/20,000,000 pixels which were significantly reduced by 36% (*p* < 0.01) compared with that of the AE group (Figure 6).

Overall, our results indicate that HTD suppresses epidermal inflammation by reducing the infiltration of inflammatory cells and modulating p-ERK, p-JNK, and p-mTOR expression.

### 3.6. Genistein as an ECS Modulator

Genistein was reported to maintain the homeostasis of the skin barrier by increasing the lipid barrier integrity in NC/Nga mice induced AD [51]. In particular, genistein has been shown to affect ECS by blocking FAAH to inhibit cellular uptake of AEA [52]. In our previous studies, high-performance liquid chromatography (HPLC) analysis of Douchi extract showed that the ratio of genistein was the highest among the active ingredients of Douchi extract [26]. Thus, we confirmed the lipid barrier formation effect of genistein, which acts as an ECS modulator, and compared the effect with PEA, one of the ECS agonists.

**Figure 7:**
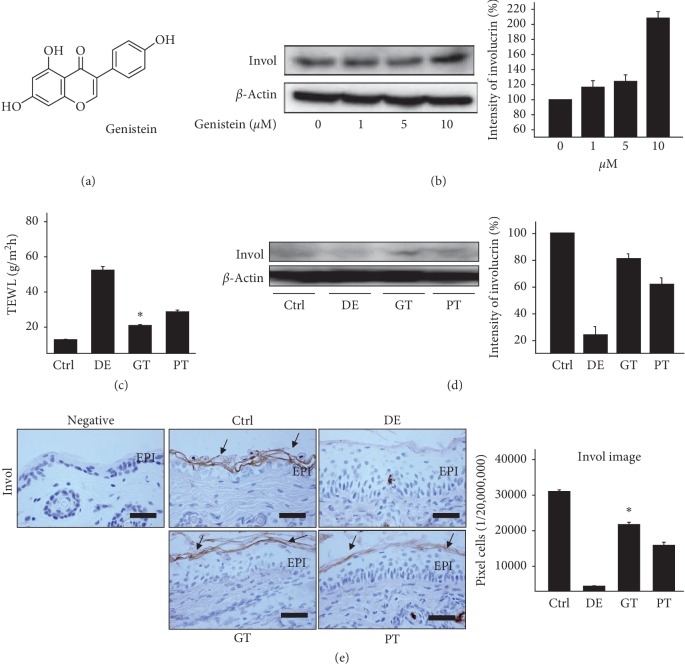
The effect of genistein as ECS modulator. (a) Structure of genistein. (b) Expression of involucrin was detected by western blotting using genistein. *β*-Actin protein was used as an internal control. (c) Effect of genistein on TEWL. Genistein significantly moisturized the skin compared to the DE group (*p* < 0.01). (d) In western blotting result, the involucrin expression was markedly increased in the GT group (*p* < 0.01). (e) The expression of involucrin (arrow indicates light brown) was significantly increased in the GT group as compared with the DE group (*p* < 0.01). The expression of involucrin in the PT group was lower than that in the GT group (bar size, 50 *μ*m). ^*∗*^*p* < 0.01 compared with the DE group. Ctrl: normal, DE: dermatitis-induced with no treatment, GT: dermatitis-induced with genistein treatment, PT: dermatitis-induced with PEA treatment, EPI: epidermis, Invol: involucrin, and TEWL: transepidermal water loss.

To estimate the effect of genistein on the formation of the skin lipid barrier, we performed western blot and measured expression of the levels of involucrin. As a result, treatment with genistein increased the expression level of involucrin in a concentration-dependent manner (Figure 7(b)).

The effects of genistein and PEA were compared by measuring the expression of involucrin using western blot and immunohistochemistry in dermatitis-induced animal models.

Western blotting results showed that the involucrin expression was markedly increased in the GT group. The expression of involucrin in the PT group was lower than that in the GT group (Figure 7(d)).

The levels of involucrin the SC were markedly reduced in the DE group, whereas the levels of involucrin in the GT group were increased by 402% (*p* < 0.01) compared with those in the DE group. Moreover, the expression of involucrin in the PT group was 15,969 ± 821/20,000,000 pixels which were significantly lower by 27% (*p* < 0.01) compared to the GT group of 21,743 ± 717/20,000,000 pixels (Figure 7(e)).

Moreover, these results were consistent with those of TEWL. Genistein significantly moisturized the skin compared to the DE group. The levels of TEWL in the GT group were decreased by 60% (*p* < 0.01) compared with those of the DE group (Figure 7(c)).

Overall, our results indicate that genistein, one of the active ingredients of Douchi extract, acted as an ECS modulator and showed a better lipid barrier formation effect than PEA.

## 4. Discussion

The epidermis maintains the homeostasis of the skin barrier by constantly repeating the proliferation, differentiation, and cornification of new epidermal cells [53]. Epidermal homeostasis means that keratinocytes undergo differentiation to form a skin barrier called the SC and to continuously maintain the permeability barrier function [54]. Skin diseases such as AD are caused by abnormalities in the physiological balance required to maintain epidermal homeostasis [55]. In particular, the ECS is intervened in the regulation of skin homeostasis by modulating cell growth, differentiation, and immune and inflammatory responses [13]. Recent evidence revealed that inadequate operation of the ECS can affect skin pathologies, such as skin barrier dysfunction in AD [56]. Therefore, the ECS is emerging as a new treatment target for various skin diseases.

The ECS consists of cannabinoid receptors (CBRs), their endogenous ligands, and the enzymes involved in the synthesis and degradation of CBs [57]. Two major CBRs are CB1 and CB2, and recently another G protein-coupled cannabinoid receptor, GPR55, has emerged as a type 3 CBR [58]. Several studies have shown that CB1 agonists relieve inflammatory symptoms [59] by downregulating mast cell activation [60] and decreasing keratinocyte-derived proinflammatory mediators [61]. In addition, CB2 agonists suppress skin inflammation by inhibiting inflammatory cell migration [62], and GPR55, which is found in mast cells, has anti-inflammatory effects by inhibiting mast cell-mediated release of nerve growth factor and reducing angiogenesis [63]. In our study, the results indicate that HTD generates ECS components such as CB1, CB2, and GPR55. Based on this result, we suggest that HTD alleviates AD symptoms by intervening in the ECS.

As previously mentioned, the keratinocyte differentiation markers, filaggrin, involucrin, and loricrin, have a crucial function by forming the CE to maintain the epidermal barrier [64]. Filaggrin plays an important role in maintaining skin surface hydration by protecting SC integrity and the generation of natural moisturizing factors [65]. Filaggrin expression has been reported to be reduced in AD [66], and filaggrin mutations have been reported to cause skin inflammation, causing skin barrier damage in AD and promoting IgE sensitization through the damaged skin barrier [67]. Thus, a filaggrin deficiency vitally related to the pathogenesis of AD [68]. Additionally, the levels of loricrin and involucrin have been reported to be lower in AD than in healthy skin [69], and the reduced expression of loricrin and involucrin was found to be downregulated by Th2 cytokines through the STAT-6 signaling pathway [36]. In addition, several studies have been conducted on the role of the ECS in the control of epidermal differentiation [70]. Recently, the specific role of CB1 and CB2 in keratinocyte differentiation has been reported to have opposite effects in the regulation of epidermal barrier permeability and keratinocyte differentiation [71]. AEA mediates CB1-dependent transcriptional processes related to epidermal differentiation and skin development [14]. According to our results, HTD increased the expression of not only CB1, CB2, and GPR55 but also filaggrin, involucrin, and loricrin. Moreover, HTD improved Lass2 expression and reduced PKC expression. Lass2 is the most widely distributed ceramide synthase in the human body and catalyzes the synthesis of heavy chain ceramides [39]. Ceramides are an important factor in determining the water retention property and epidermal barrier integrity [72]. In AD skin lesions, reduced ceramide levels were observed [6]. Reduced ceramides affect PKC activation in AD, leading to the production of inflammatory cytokines and barrier disruption [73]. These findings mean that HTD reduced the PKC activation observed in AD by increasing ceramide synthesis. Based on these results, we suggest that HTD enhances epidermal barrier integrity by promoting keratinocyte proliferation from stratum granulosum (SG) to SC and lipid barrier formation by intervening in ECS.

E-cadherin expression was downregulated on keratinocyte surfaces in the lesional skin of AD [74], and dissolution of the tight junctions that are important for maintaining the barrier function of the epidermis was followed by downregulation of E-cadherin expression [75]. Oxidative stress directly affects epidermal keratinocytes and induces intracellular changes, resulting in edema, spongiosis, and destruction of SC [76]. GPx4 is an antioxidant enzyme that recovers oxidative damage and is increased in expression as a defense mechanism against oxidative stress [77]. Recently, it was revealed that GPx4 regulates the release of apoptotic proteins and plays a critical role in cellular homeostasis through apoptosis [43]. In GPx4 deletion mouse experiments, epidermal hyperplasia, dermal inflammatory infiltration, and reduced keratinocyte adhesion were induced [78]. In our study, HTD maintained the epidermal structure by regulating the expression of E-cadherin and GPx4.

MAPKs, including ERK and JNK, are essential signals for the production of cytokines such as IL-4 and TNF-*α* [79] and regulate the inflammatory response through nuclear factor-kappa B (NF-κB) activation [80]. IL-17, a proinflammatory cytokine, decreases the expression of filaggrin and involucrin in AD via the MAPKs signal pathway [81]. In our study, HTD also modulated the inflammatory response by blocking the MAPK signaling pathway by inhibiting the phosphorylation of ERK and JNK.

In addition, we confirmed the effect of genistein, one of the active ingredients to strengthen our results. In western blotting, genistein increased the expression level of involucrin in a concentration-dependent manner. In comparison with PEA, the expression of involucrin was significantly increased. Thus, the HTD with Douchi extract acts an ECS modulator and showed a better lipid barrier formation effect than PEA.

In this study, we demonstrated the effects of HTD on AD using a *Df*E-induced AD model. HTD improved external pathological features and AD skin scores and reduced angiogenesis and epithelium hyperplasia. HTD also inhibited IL-4 expression. Furthermore, orally administered Douchi extracts as HTD significantly generated ECS components, such as CB1, CB2, and GPR55, and lipid barrier formation and suppressed hyperplasia and infiltration of inflammatory cells. In particular, the HTD-mediated operation status of ECS was higher than that of the Ctrl group. Therefore, the HTD-mediated alleviation of AD-induced skin lesions was observed *in vivo*.

In conclusion, HTD can alleviate symptoms of AD such as erythema, bleeding, scarring, erosion, and excoriation by maintaining skin homeostasis, improving skin barrier formation, and downregulating inflammation, through ECS intervention. Therefore, this study shows the potential of HTD for the treatment of AD as a prophylactic treatment to maintain homeostasis through epidermal structure improvement.

## 5. Conclusions

In this study, we demonstrated that HTD effectively suppressed the development of symptoms of AD using *Df*E-induced NC/Nga AD mice. These results suggest that HTD intervenes in the ECS to maintain skin homeostasis, thereby maintaining the epidermal structure and downregulating inflammation.

## Data Availability

The data used to support the findings of this study are available from the corresponding author upon request.

## Abbreviations

HTD: Hataedock
AD: Atopic dermatitis
*Df*E: *Dermatophagoides farinae* extract
CB: Cannabinoid receptor type
GPR55: G protein-coupled receptor 55
Lass2: Longevity assurance homolog 2
GPx4: Glutathione peroxidase 4
CD1A: Cluster of differentiation 1a
p-ERK: Phosphorylated extracellular signal-related kinase
p-JNK: Phosphorylated c-Jun amino-terminal kinase
p-mTOR: Phosphorylated mammalian target of rapamycin
PEA: Palmitoylethanolamide
SC: Stratum corneum
ECS: Endocannabinoid system
CBs: Cannabinoids
AEA: Anandamide
2-AG: 2-Arachidonoylglycerol
FAAH: Fatty acid amide hydrolase
TEWL: Transepidermal water loss
M/T: Masson's trichrome
P/T: Phloxine-tartrazine
PKC: Protein kinase C
TUNEL: Terminal deoxynucleotidyl transferase dUTP nick end labeling
PVDF: Polyvinylidene difluoride
ANOVA: One-way analysis of variance
CE: Cornified cell envelope
SP: Stratum spinosum
SB: Stratum basale
MAPKs: Mitogen-activated protein kinase
AP-1: Activator protein 1
CBRs: Cannabinoid receptors
CBs: Cannabinoids
SG: Stratum granulosum
IL: Interleukin
TNF-*α*: Tumor necrosis factor-alpha.
